# Crystallization and preliminary X-ray diffraction analysis of YejM from *Salmonella typhimurium*: an essential inner membrane protein involved in outer membrane directed cardiolipin transport

**DOI:** 10.12688/f1000research.8647.2

**Published:** 2017-12-11

**Authors:** Uma Gabale, Gene Qian, Elaina Roach, Susanne Ressl

**Affiliations:** 1 Molecular and Cellular Biochemistry Department, Indiana University Bloomington, Bloomington, IN, 47405, USA

**Keywords:** Salmonella typhimurium, cell growth, outer membrane, membrane protein, YejM, YejL, cardiolipin

## Abstract

*Salmonella* *typhimurium* is responsible for over 35% of all foodborne illness related hospitalizations in the United States. This Gram-negative bacterium possesses an inner and an outer membrane (OM), the latter allowing its survival and replication within host tissues. During infection, OM is remodeled by transport of glycerophospholipids across the periplasm and into the OM. Increased levels of cardiolipin in the OM were observed upon PhoPQ activation and led to the discovery of YejM; an inner membrane protein essential for cell growth involved in cardiolipin binding and transport to the OM. Here we report how YejM was engineered to facilitate crystal growth and X-ray diffraction analysis. Successful structure determination of YejM will help us understand how they interact and how YejM facilitates cardiolipin transport to the OM. Ultimately, *yejm*, being an essential gene, may lead to new drug targets inhibiting the pathogenic properties of *S. typhimurium*.

## Introduction

*Salmonella typhimurium* is a Gram-negative bacterium responsible for over 35% of all foodborne illness-related hospitalizations in the United States ( Painter *et al.*, 2013). *S. typhimurium* possesses an additional outer membrane (OM) with an asymmetric lipid composition, that serves as a barrier to the environment allowing its survival and replication within host tissues ( Dalebroux & Miller, 2014; Needham & Trent, 2013; Pagès *et al.*, 2008). Mechanisms for the transport and assembly of OM lipopolysaccharides, proteins, and exopolysaccharides have been defined ( Dong *et al.*, 2006; Dong *et al.*, 2014; Hagan *et al.*, 2011; Whitfield & Trent, 2014); however, the transport of glycerophospholipids across the periplasm and insertion into the inner-leaflet of the OM is not well understood.

**Figure 1.**
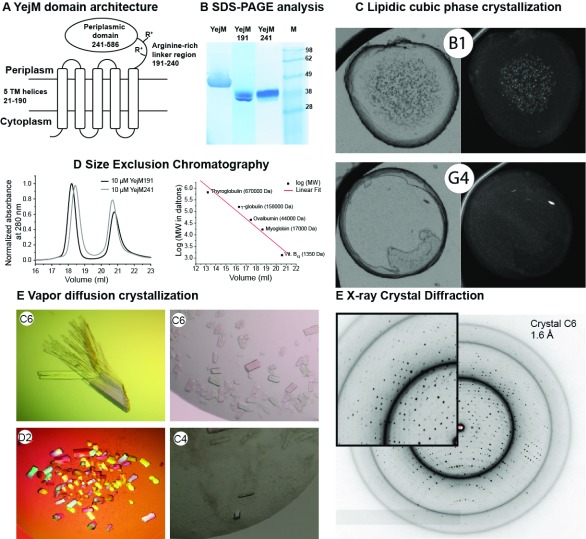
YejM architecture, purification, crystallization and diffraction data. **A**) YejM protein domain architecture. **B**) SDS-PAGE analysis of purified protein samples. **C**) LCP crystallization droplets of full length YejM mixed with Monoolein. Crystals are shown in light microscopy image (left) and corresponding UV image (right). B1 and G4 refer to conditions from MemGold crystal screen 1. **D**) Crystals of YejM241 under various conditions. **E**) Diffraction image of a YejM241-586 crystal from condition C6.

Interestingly, increased levels of cardiolipin in *S. typhimurium* OM were observed upon PhoPQ regulator activation ( Dalebroux *et al.*, 2014). Recently the inner membrane protein YejM (also known as PbgA) was shown to bind cardiolipin and be involved in OM formation ( Dalebroux *et al.*, 2015). Furthermore, YejM is known to be an essential gene in *E. coli* and was shown to be involved in intrinsic multidrug resistance ( De Lay & Cronan, 2008; Duo *et al.*, 2008). YejM is comprised of 586 amino acids forming five predicted N-terminal transmembrane helices, followed by an arginine-rich periplasmic random coil linker region, and a C-terminal periplasmic domain ( Figure 1A), and it was shown that YejM associates as a tetramer in solution ( Dalebroux *et al.*, 2015). The arginine-rich linker region and periplasmic globular domain of YejM were demonstrated to bind cardiolipin and are required for OM remodeling and cell growth ( Dalebroux *et al.*, 2015). The molecular mechanism of the interplay between PhoPQ system and YejM, and how cardiolipin molecules are transported to the OM, need further structural and functional investigation.

Here we report crystal growth and present successful crystallization conditions for full-length YejM from *Salmonella typhimurium* using lipidic cubic phase crystallization and how the periplasmic domain of YejM was engineered to facilitate crystallization, and present preliminary X-ray diffraction analysis of the construct YejM241-586. Future successful structure determination of YejM will help us to understand cardiolipin transport to the OM and may lead to new drug targets inhibiting the pathogenic properties of *S. typhimurium*.

## Results

### Full-length YejM crystallized using lipidic cubic phase

Full-length YejM was expressed and purified ( Figure 1B, first lane) as described in ( Dalebroux *et al.*, 2015), and concentrated to 15 mg/ml in the presence of 0.01% DDM. Micro crystals appeared in many conditions after two weeks. Figure 1C shows examples of LCP boli with micro crystals as judged by UV microscopy. Further work on full-length YejM will be aimed to optimize current crystal conditions and optimize detergents for LCP and regular vapor diffusion crystallization.

### Specific construct development was needed to achieve YejM crystal growth

We expressed and purified periplasmic construct YejM191-586 as described in ( Dalebroux *et al.*, 2015). The original construct YejM191-586 failed to crystallize and showed a degradation product after electrophoresis in the SDS polyacrylamide gel ( Figure 1B, second lane). To prevent degradation, reduce flexible protein parts and remove positively charged arginine clusters, and increase the chance for crystal growth, we deleted the linker region A191 to E240 in the YejM191-586 construct, resulting in YejM241-586 ( Figure 1B, third lane). Both constructs eluted as well-defined peaks when applied to size exclusion chromatography (SEC) ( Figure 1D). A clear shift in size between both constructs was observed in SEC when performed timely after affinity chromatography. When compared to the standards, both constructs eluted at later volumes than expected for their size ( Figure 1D). Initial crystals of YejM241-586 appeared after one week incubation at 18°C under different conditions; e.g. needle clusters in 2.8 M sodium acetate trihydrate pH 7.0, 0.1 M BIS-TRIS propane pH 7.0 (Hampton SaltRx condition A2, Hampton Research), needle clusters in 2.8 M sodium acetate (Hampton Index condition B12, Hampton Research), and rhombohedral crystals appeared in 3.5 M sodium formate pH 7.0 (Hampton Index HR conditions C1, Hampton Research). These early hit crystals diffracted poorly, only up to 6Å and optimization and up-scaling of the crystallization setup from a 96 well format to a 24 well format did not improve the diffraction quality. Further screening using (Hampton PEGRx HT screen, Hampton Research) resulted in new crystal forms grown in condition C6 (0.1 M HEPES pH 7.5, 12% w/v polyethylene glycol 3,350) and C4 (0.1 M Citric acid pH 3.5, 25% w/v polyethylene glycol 3,350). Screening around these two conditions led to crystals in a condition consisting of (0.1 M citric acid pH 4, 18% w/v polyethylene glycol 3,350 ( Figure 1C). YejM241-586 crystals grown in condition C6 diffracted well, up to 1.6 Å ( Figure 1E). Data indexing and scaling with XDS ( Kabsch, 2010) and further analysis with AIMLESS ( Evans, 2006) resulted in a data set up to 1.8Å resolution and good overall statistics ( Table 1).

**Table 1.** Diffraction data statistics of crystal grown in condition C6 ( Figure 1C).

Diffraction source	ALS BL 4.2.2
Wavelength (Å)	1.001
Temperature (K)	100
Detector	CMOS
Crystal-to-detector distance (mm)	200
Rotation range per image (°)	0.106
Total rotation range (°)	190
Space group	P 1 2 1
a, b, c (Å)	82.58, 86.85, 89.03
α, β, γ (°)	90.00, 115.39, 90.00
Resolution range (Å)	59.01–1.8 (1.9–1.8)
Total No. of reflections	315601 (30716)
No. of unique reflections	94930 (9801)
Completeness (%)	91.5 (64.8)
Multiplicity	3.3 (3.1)
<I/σ(I)>	11.2 (2.2)
R _meas_	8.7 (80.4)

## Conclusions

Here we report successful crystallization using protein engineered specifically to enable crystal growth of YejM. Our initial X-ray data analysis of YejM241-586 crystals suggests a dimer assembly of the periplasmic domain, therefore the membrane domain is very likely needed to form the YejM tetramer. We also aim to solve the crystal structures of YejM alone and with bound cardiolipin. Ultimately these structures will help in understanding where and how cardiolipin binds to YejM, and whether YejM’s architecture is that of a transporter or channel.

## Material and methods

### Cloning, expression and purification of YejM

Initial clones of full-length YejM 1-586 (YejM) (Uniprot ID P40709) in pBAD24 and the periplasmic domain of YejM 191-586 in pET28a plasmid are described in ( Dalebroux *et al.*, 2015). We used forward primer YejM241-586 5'-ccgcgcggcagccatatggctagcgcggtctccgttcagtacccg- 3' and reverse primer YejM241-586 5'-gcgggtactgaacggagaccgcgctagccatatggctgccgcgcgg- 3' to create a shorter construct of the periplasmic domain lacking the linker region resulting in YejM 241-586. Purification of YejM, YejM 191-586, and YejM 241-586 was performed as described previously ( Dalebroux *et al.*, 2015). Samples used for subsequent crystallization experiments were further purified by SEC using a Superose 6 increase 10/300 GL column (GE Healthcare) in buffer containing 50 mM Tris pH 8.0, and 150 mM NaCl. SEC buffer for YejM contained 20 mM HEPES, pH 7.5, 150 mM NaCl, and 0.02% Dodecyl-β-D-Maltopyranoside (DDM). The concentration of DDM was kept right above the critical micelle concentration throughout all subsequent experiments. YejM and YejM241-586 SEC peak fractions were pooled and concentrated with 30 kDa NMWL centricon (Millipore) to 12 mg/ml and up to 50 mg/ml, respectively. The purity of the samples was judged by polyacrylamide gel electrophoresis ( Figure 1B).

### Crystallization of full-length YejM using lipidic cubic phase crystallization

YejM (15 mg/ml) and Monoolein (Hampton Research) were mixed at a 1:1.5 ratio at 23°C in a coupled Hamilton syringe using a LCP Mixer Station (ARI) until LCP mixture appeared clear. LCP crystallization screens were set up using a Gryphon robot (ARI) pipetting 50 nl LCP boli and 500–800 nl precipitant solution of the following screens: MemGold 1 and 2 (Molecular Dimensions), MembFac (Hampton Research), and JBScreen Membrane (Jena Bioscience). LCP crystallization was set up using LaminexTM plates (Molecular Dimensions).

### Crystallization of the periplasmic domain of YejM

Vapor diffusion crystallization of YejM 241-586 (5–50 mg/ml) was set up using 96-well crystallization plates (Hampton Research) with a Phoenix robot (ARI). Various sparse matrix screens were used to set up sitting drops with a drop size between 500 nl to 1 µl. Crystallization plates were incubated at 20°C and monitored for crystal growth in a Minstrel ^TM^ HT crystal imaging and detection tower (Rigaku). Optimal crystal growth was obtained at a protein concentration of 4mg/ml.

### Data collections and processing of YejM 241-586 crystals

Crystals were harvested using Litho loops (Molecular Dimensions) and Nylon loops (Hampton Research), submerged into paraffin and blotted until no phase separation was visible between paraffin and the excess crystallization solvent. Diffraction data of YejM241-586 crystals were collected at the Advanced Light Source beamline 4.2.2 in Berkeley CA at 100K, using an oscillation of 0.1–0.2° per image. Diffraction data were processed using iMosflm ( Powell *et al.*, 2013) or XDS ( Kabsch, 2010) and scaled with Scala ( Evans, 2006).

## Data availability

Raw diffraction data images were uploaded to the Coherent X-ray Imaging Data Bank ( http://cxidb.org/id-42.html) and are available under CXIDB ID 42, DOI 10.11577/1252489 ( Gabale *et al.*, 2016).

## Supplementary material

**Supplementary File 1. Supplementary Text and Experiments**

Click here to access the data.

**Supplementary Figure 1. Cellular fractionation.**

**Supplementary Figure 2. Native PAGE showing controls and mixtures of A: YejM191-586 with YejL and B: YejM241-586 with YejL.**

**Supplementary Figure 3. SEC-MALS of YejM241-586 and YejL.**
