# Supplementary material for: Crystallization and preliminary X-ray diffraction analysis of YejM from Salmonella typhimurium: an essential inner membrane protein involved in outer membrane directed cardiolipin transport
Source: F1000Res. 2017 Dec 11;5:1086. Originally published 2016 Jun 2. [Version 2] doi: 10.12688/f1000research.8647.2 (PMC5728191; doi:10.12688/f1000research.8647.2)
Supplement: Supplementary file 1 [file f1000research-5-14449-s0001.tgz › 6319fe56-b34d-4eb0-bdd8-3cd823e5e831.pdf]

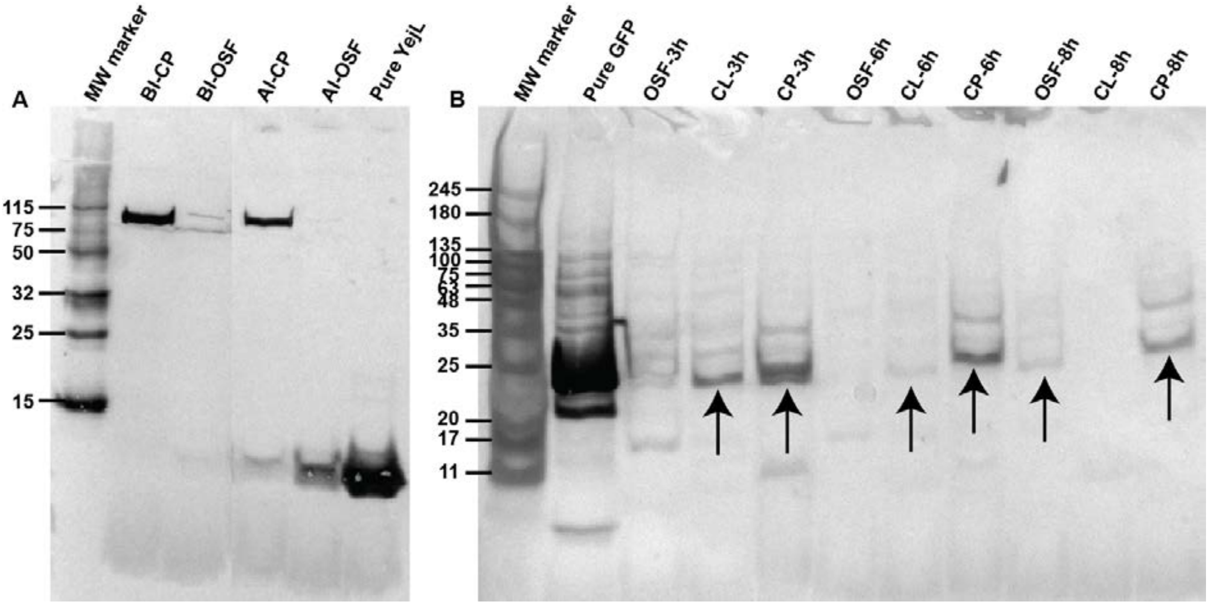

Supplementary Figure 1: Cellular fractionation.
A) YejL overexpression in *E. coli* BL21(DE3) cells at 37 ^{O}C. Molecular weight (MW) marker: Smart His-Tagged Protein Standard (Genscript; catalog no. MM0904); BI-CP: Before induction - cell pellet; BI-OSF: before induction - osmotic shock fluid (OSF); AI-CP: 4 h After induction -CP; AI-OSF: 4h after induction - OSF; Pure YejL: purified YejL control. YejL (10 kDa) is detected in BI-OSF of non-induced cells, and in both, cell pellet and OSF after induction (AI-CP and AI-OSF. The high molecular weight bands observed (~115 kDa) in both, BI and AI cell pellets might originate from non-specific binding of the anti-6His antibody (AbCam cat#AB49746) to chaperones.
Cell growth: To determine whether YejL was expressed and/or present in cytoplasm or periplasm, we overexpressed YejL in *E. coli* BL21(DE3) cells. After induction, cultures were grown at 37 ^{O}C. Cells were harvested from 1 ml culture before induction and at each hour after induction until 4 hours and frozen at -20 ^{O}C until further processing. To prepare OSF, frozen cells were thawed on ice and 50 µL of sucrose buffer (30 mM Tris, pH 8.0, 20 % sucrose, 1 mM EDTA) was added to each cell pellet. Cell suspensions were incubated on ice for 10 min and centrifuged at 8000 rpm for 10 min. After discarding the supernatant, 50 µL of 5 mM $MgSO_4$ was added to each cell pellet, mixed and incubated for 10 min. Centrifuging at 8000 rpm for 10 min yielded clear OSF, which contained periplasmic fraction. Cell pellet from centrifugation was resuspended in 50 µL 1X PBS for SDS PAGE analysis. B) Expression of YejL coupled with Yellow Fluorescent Protein (YejL-YFP =37 kDA) in *E. coli* strain SX1992 at 37 ^{O}C. MW marker: BLUEstain protein ladder 11-245 kDa (GoldBio catalog no. P007-500); GFP: Green Fluorescent Protein (control); OSF-3h: osmotic shock fluid time-point 3h; CL-3h: cell lysate after separating OSF time-point 3h; CP-3h: cell pellet after separating OSF and lysing cells time-point 3h. Samples repeat for time-points as indicated. Although the anti-GFP antibody (Abcam cat#AB6661) shows a high amount of non-specific binding, we interpret YejL-YFP as being detected in samples CL-3h, CP-3h, CL-6h, CP-6h, OSF-8h and CP-8h (indicated by arrows), suggesting that YejL is present in the OSF after 8h and cell growth at 37 ^{O}C.

Cell growth: The strain SX1992 was grown in LB medium containing 30 µg/ml chloramphenicol at 37 ^{O}C. Cells were harvested at different time points (3 h, 6 h, 8 h) and frozen at -20 ^{O}C until further processing. Preparation of cell lysate: All steps were carried out at 4 ^{O}C unless noted otherwise. Cells from 1 ml culture previously treated with sucrose buffer to obtain OSF were lysed by incubation with lysis buffer (1X PBS containing 1 mM EDTA, 1 mM PMSF, cocktail protease inhibitors (GoldBio), and 0.5

mg/ml lysozyme). Cell lysate and pellet were separated by centrifugation at 6400g for 10 min. OSFs, cell lysates and cell pellets from each time point were analyzed by SDS PAGE followed by Western blot to identify presence of YejL or YejL-YFP in each fraction. Polyacrylamide gel electrophoresis and Western Blot: Samples were resolved on a 10% SDS polyacrylamide gel and transferred to a nitrocellulose membrane (Invitrogen) using the iBlot® dry blotting system (Life Technologies). The membrane was blocked for 1 hour at room temperature by 3% BSA (Fisher Scientific) solution in 1X PBS (Santa Cruz Biotechnology, Inc) containing 0.05% tween 20 (Sigma-Aldrich). After three washes with 1X PBS, 20 mL of 1:2000 diluted solution of anti 6X His antibody (A) and anti-GFP antibody (B, C, and D) both conjugated with alkaline phosphatase (AP) in 1X PBS, 0.05% tween 20 were added to the membrane, respectively. Antibodies incubated overnight at 4 ^{0}C on a horizontal rotator. The antibody solution was discarded and the membrane was washed three times with 1X PBS. To detect the AP-linked antibodies, one tablet of SIGMA FAST™ 5-Bromo-4-chloro-3-indolyl phosphate/Nitro blue tetrazolium (BCIP/NBT) (Sigma-Aldrich) was dissolved in 10 ml of deionized water and poured on the membrane, color developed in about 1-4 minutes.
