# Supplementary material for: Crystallization and preliminary X-ray diffraction analysis of YejM from Salmonella typhimurium: an essential inner membrane protein involved in outer membrane directed cardiolipin transport
Source: F1000Res. 2017 Dec 11;5:1086. Originally published 2016 Jun 2. [Version 2] doi: 10.12688/f1000research.8647.2 (PMC5728191; doi:10.12688/f1000research.8647.2)
Supplement: Supplementary file 2 [file f1000research-5-14449-s0002.tgz › 654c2984-5f92-43b4-9a33-454cd9a505e1.pdf]

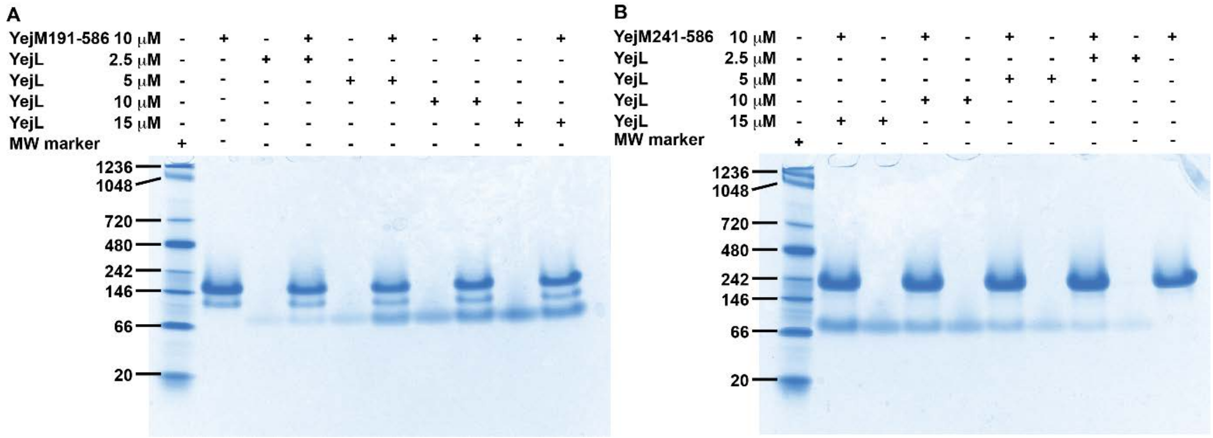

Supplementary Figure 2: Native PAGE showing controls and mixtures of A: YejM191-586 with YejL and B: YejM241-586 with YejL.

MW marker: Novex NativeMark Unstained Protein Standard (Invitrogen; catalog no. LC0725). 10 μM of YejM191-586 or YejM241-586 was mixed with varying concentrations (2.5, 5, 10, and 15 μM) of YejL separately.

A) YejM191 shows clear degradation into two bands at slightly above and below the 146 kDa mark. No population of increased size was detected with increasing amounts of YejL. B) YejM241 runs as a hexamer of ~242kDa showing no signs of degradation. No population of increased size was detected with increasing amounts of YejL. In both gels YejL runs at a size slightly higher than 66kDa, indicating a YejL hexamer or higher. Samples were incubated at 4 °C for minimum 1 hr, mixed with equal volume of 2X sample buffer (Novex™ Tris-Glycine Native Sample Buffer, Invitrogen, catalog no. LC2673) to a total individual sample volume of 40 μL. The samples were loaded on Novex™ 4-20% Tris-Glycine Mini Gel (Invitrogen, catalog no. XP04200BOX) and electrophoresis was carried out in a Mini Gel Tank (Invitrogen, catalog no. A25977) filled with Novex™ Tris-Glycine Native Running Buffer (Invitrogen catalog no. LC2672) at 4 °C at 75 V for ~120 min. After completion of electrophoresis, the gels were fixed by heating for 1 min in a microwave oven in Solution 1 containing 10% acetic acid and 50 % ethanol, cooled for 5 min, Solution 1 was discarded and gel was stained by heating for 1 min in the microwave oven in Solution 2 containing 5 % ethanol, 7.5% acetic acid, and 0.1% Coomassie Brilliant Blue G-250 and kept at RT on rocker until protein bands appeared.
