# Supplementary material for: Crystallization and preliminary X-ray diffraction analysis of YejM from Salmonella typhimurium: an essential inner membrane protein involved in outer membrane directed cardiolipin transport
Source: F1000Res. 2017 Dec 11;5:1086. Originally published 2016 Jun 2. [Version 2] doi: 10.12688/f1000research.8647.2 (PMC5728191; doi:10.12688/f1000research.8647.2)
Supplement: Supplementary file 3 [file f1000research-5-14449-s0003.tgz › 0ce73e4f-d4e1-45f6-88d9-7c7e6aaf217f.pdf]

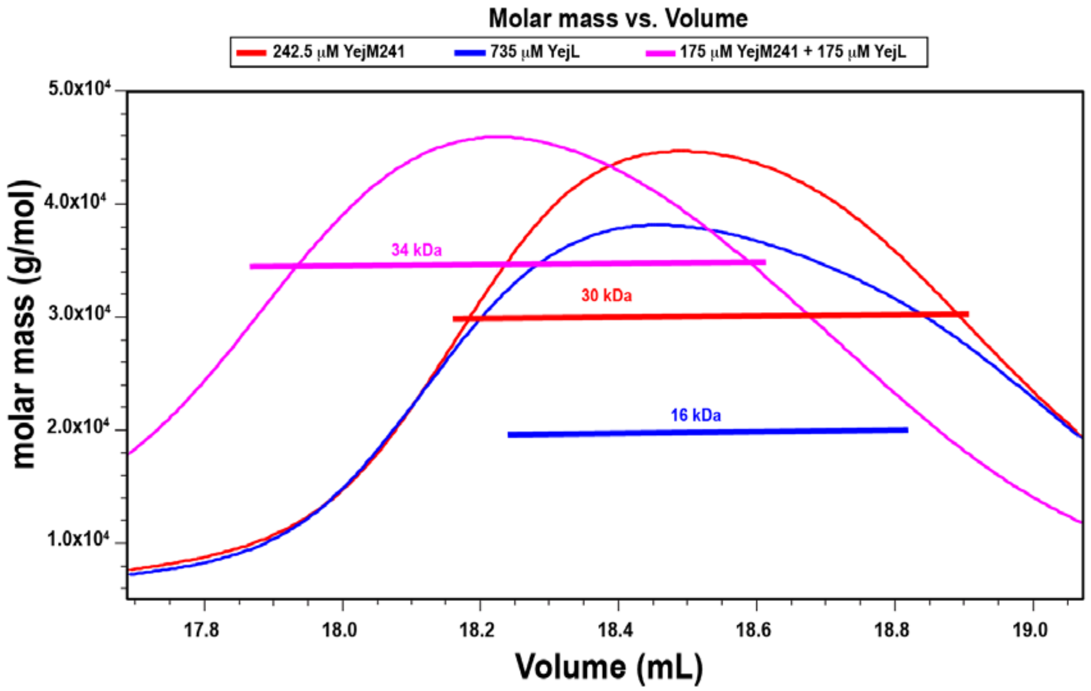

Supplementary Figure 3 SEC-MALS of YejM241-586 and YejL.
SEC-MALS studies of YejM241-586 (red line), YejL (blue line) and their 1:1 complex (magenta). 100 µL total volume of protein sample was injected onto a Superose 6 increase 10/300 GL column (GE Healthcare) attached to a multiangle light-scattering system equipped with Dawn Helios II light scattering detector and an Optilab T-rEX differential Refractive Index (dRI) detector (all Wyatt Technology Corporation). Data collection and analysis was carried out using Astra 5 software (Wyatt Technology Corporation). The software calculated protein molecular weight and concentration based on a refractive index increment dn/dc value of 0.185 mL/g. The entire experiment was carried out at 25 ^{0}C. YejL (blue line) resulted in an absolute molecular mass of 14.6 kDa (+/- 1.2 kDa), which is higher than the calculated molecular weight of 10 kDa. Yejm241-586 alone (red line) resulted in an absolute molecular mass of 29.6 kDa (+/- 0.2 kDa), which is considerably lower than the calculated molecular mass of 41.1 kDa. The mixture of YejM241-586 and YejL (magenta line) resulted in an absolute molecular mass of 32.9 kDa (+/- 1.3 kDa), which is closer to the calculated molecular weight of YejM241-586. In fact, in all our SEC-MALS experiments for YejM241-586, the highest absolute molecular mass observed was 33 kDa, independent of concentration. This corresponds with the size at which YejM241-586 runs on the SDS-PAGE and SEC (Figure 1B/D in main manuscript).
