# Supplementary material for: Crystallization and preliminary X-ray diffraction analysis of YejM from Salmonella typhimurium: an essential inner membrane protein involved in outer membrane directed cardiolipin transport
Source: F1000Res. 2017 Dec 11;5:1086. Originally published 2016 Jun 2. [Version 2] doi: 10.12688/f1000research.8647.2 (PMC5728191; doi:10.12688/f1000research.8647.2)
Supplement: Peer review history

# Peer review: Referee response for version 2

**DOI:** 10.5256/f1000research.14449.r28934

**Published:** 2017-12-12

**Authors:** William T. Doerrler

**Affiliations:** 1 Department of Biological Sciences, Louisiana State University, Baton Rouge, LA, USA

In this update, the authors have provided helpful data suggesting a periplasmic localization of YejL and clearer native-PAGE gels.

I have read this submission. I believe that I have an appropriate level of expertise to confirm that it is of an acceptable scientific standard.

# Peer review: Referee response for version 1

**DOI:** 10.5256/f1000research.9303.r14811

**Published:** 2016-07-06

**Authors:** Yihua Huang

**Affiliations:** 1 National Laboratory of Biomacromolecules, National Center of Protein Science-Beijing, Institute of Biophysics, Chinese Academy of Sciences, Beijing, China

In this manuscript, Gabele *et al*. reported the structure of YejL and preliminary X-ray crystallographic characterization of the periplasmic domain (Residues 241-586) of YejM, an inner membrane protein potentially responsible for transport of glycerophospholipid cardiolipin. The authors also tested interaction between YejL and YejM using blue native gel electrophoresis and size exclusion chromatography. In general, it is an interesting study, yet the following issues need to be addressed before accepted for indexation:

  

1. It is not proper to claim that YejL is a ligand of YejM. “Ligand” is dedicated for “receptors” only. Also, there is no strong genetic evidence to show that YejL is involved in cardiolipin transport.
2. The authors claimed that YejM is a dimer based on its periplasmic domain, and probably, also because of YejL forming a dimer. This is not clear as the authors did not study the oligomerization state of the full-length YejM . Other than this, when running SEC on superpose 6, the authors did not include a standard marker for reference. It ishard to judge if the periplasmic domain of YejM forms a dimer in solution.
3. The author believed that the negative charge surfaces of YejL might bind with the arginine-rich loop of YejM. Why the fragment of YejM (241-586) that seems to not include the arginine-rich loop still binds YejL ? Please explain.

I have read this submission. I believe that I have an appropriate level of expertise to confirm that it is of an acceptable scientific standard.

# Response

**Published:** 2017-11-28

**Authors:** Susanne Ressl

**Affiliations:** Indiana University at Bloomington

1.) We apologize for the incorrect use of terminology and would rather term YejL as potential interaction partner of YejM.

2.) We carefully checked within the manuscript what section/sentence could have caused this reviewer’s understanding that we claimed YejM to be a dimer. We found the following sentence in the “Conclusions” section of our originally submitted manuscript: *“* *Our initial X-ray data analysis of YejM241-586 crystals suggests a dimer assembly of the periplasmic domain, therefore the membrane domain is very likely needed to form the YejM tetramer”.* **

In this sentence, we suggested the existence of a periplasmic domain YejM241-586 dimer based on our analysis of the crystallographic unit. In this analysis the content of the asymmetric unit that builds the crystal resulted in a dimer of YejM 241-586, and therefore we proposed it to be the likely physiologically relevant state of the periplasmic domain alone. From the SEC-MALS studies in (Dalebroux *et al.* 2015) that full-length YejM forms a tetramer when purified with the detergent DDM. Therefore we reasoned in the above stated sentence that further oligomerization into a tetramer must be facilitated by the trans-membrane domain. Whether is exists as a dimer or a monomer either in the native membrane, in the presence of specific lipids or purified using a different detergent is a matter of current investigation by other laboratories (Miller and Dalebroux labs).

3.) Indeed, the arginine-rich linker region of YejM would be the most obvious binding region for YejL. However, we cannot exclude the possibility that YejL binds to solvent-accessible positively charged residues on the YejM241-586 surface.

# Peer review: Referee response for version 1

**DOI:** 10.5256/f1000research.9303.r14132

**Published:** 2016-06-03

**Authors:** William T. Doerrler

**Affiliations:** 1 Department of Biological Sciences, Louisiana State University, Baton Rouge, LA, USA

Here, the authors report the expression, purification and isolation of diffractable crystals of the periplasmic domain of the essential salmonella protein YejM, putatively involved in transport of cardiolipin to the outer membrane. They also report interaction between the periplasmic domain of YejM and the small protein YejL produced from a gene immediately upstream and likely cotranscribed with YejM.

 YejM was identified by a screen for mutants defective in PhoPQ mediated outer membrane remodeling and was renamed PbgA (PhoPQ barrier gene A). The regions of the protein and their proposed functions are as follows: 1-190, transmembrane domain of the inner membrane; 191-240, arginine-rich linker that binds to cardiolipin and 240-586, the periplasmic domain seemingly required for interaction with OM. The authors report isolation of crystals of the YejM241-596 periplasmic domain and interaction of YejL with YejM241-586 and YejM191-586 truncated mutants. Sadly, the inability to crystalize YejM191-586 will forestall attempts to examine cardiolipin-YejM interactions.

 This work represents a preliminary attempt to understand at the molecular level an extremely interesting process required for Salmonella virulence namely OM lipid remodeling. YejM/PbgA represents a novel virulence factor and potential drug target. The importance of its identification cannot be understated.

 Major issues:

1. This reviewer questions the physiological relevance of the interaction between YejL and the YejM periplasmic domains. YejL is a 75 amino acid protein encoded by the gene immediately upstream of YejM. It is not predicted to contain a secretion signal and the PDB itself predicts it to be a soluble cytoplasmic protein. It is difficult to envision how it would interact with the periplasmic domain of YejM in a living cell. Cellular fractionation studies showing a periplasmic localization of YejL would need to accompany the data in Figure 2 in order for it to be meaningful. Genetic studies may also be useful- for example, does the YejL mutant show alterations in PhoPQ mediated survival or YejM tetramer formation?
2. The BNE gels are not convincing. Why does YejL migrate at such a large molecular weight? YejM migrates as a smear in both gels when loaded by itself. Adding YejL reduces the smear but it cannot be said that YejL alters the MW of YejM in either gel.

Minor issues:

1. Why not call YejM by its new name PbgA throughout? It would cause less confusion since the senior author was associated with the publication reporting the discovery last year.
2. Figure 1A, the membrane domain in the figure should be labeled 0-190 not 191-240. Also the “j” in “YejL structure” is partially obscured.
3. I take issue with describing YejL as a ‘ligand”. To me and most biochemists a ligand is a small molecule that interacts with a target protein (receptor) causing some kind of conformational change and usually generation of a signal of some sort. What they are describing here is a protein-protein interaction and should be referred to as such, i.e. “YejL interacts with the periplasmic domain of YejM”.

I have read this submission. I believe that I have an appropriate level of expertise to state that I do not consider it to be of an acceptable scientific standard, for reasons outlined above.

## References

[1] DalebrouxZDEdrozoMBPfuetznerRAResslSKulasekaraBRBlancMPMillerSI: Delivery of cardiolipins to the Salmonella outer membrane is necessary for survival within host tissues and virulence. *Cell Host Microbe*.2015;17(4) : 10.1016/j.chom.2015.03.003 441-51 10.1016/j.chom.2015.03.003 25856753PMC4978220

# Response

**Published:** 2017-11-28

**Authors:** Susanne Ressl

**Affiliations:** Indiana University at Bloomington

Major issues:

1.) We performed cellular fractionation studies based on the reviewer's suggeestions and present the results in the supplemental section in the updated version of the manuscript. The suggested genetic studies are beyond our laboratory’s main focus and scope of revised manuscript; however these studies are being currently performed in the laboratory of Dr. Dalebroux (personal communication).

2.) We improved the quality of our BNE experiments and show the results in the supplemental section of the updated version of the manuscript.

Minor issues:

1.) PbgA is not yet officially listed as an alternative name for YejM. Uniprot lists entries for PbgA as proteins that are Phospho-beta-glucosidases. To prevent confusion, we chose to use the official Uniprot database nomenclature of YejM (http://www.uniprot.org/uniprot/P40709).

2.) This figure is not included in the updated version of the manuscript.

3.) We agree that this was not the correct term to use and would rather term YejL as potential interaction partner of YejM.

We thank both reviewers for their helpful critique.
